# Integrative Evidence Reveals the Underestimated Vulnerability of *Abies ernestii*—An Endemic Fir in Southwest China

**DOI:** 10.3390/plants15101546

**Published:** 2026-05-19

**Authors:** Tao Chen, Tingting Wang, Shigang Li, Changyou Zhao, Liding Chen, Huanchong Wang

**Affiliations:** 1 School of Ecology and Environmental Science, Yunnan University, Kunming 650500, China; chentao_s3ca@itc.ynu.edu.cn (T.C.); wangtingting_qgtw@stu.ynu.edu.cn (T.W.); shi-gangli@mail.ynu.edu.cn (S.L.); 2 Southwest United Graduate School, Kunming 650092, China; 3 Management Bureau of Yunnan Jiaozishan National Natural Reserve, Kunming 651515, China; gwhzcy@126.com; 4 State Key Laboratory of Vegetation Structure, Function and Construction (VegLab), Kunming 650500, China

**Keywords:** *Abies ernestii*, biodiversity conservation, climate change adaptation, endemic species, phylogeny, potential distribution, taxonomy

## Abstract

Endangered montane endemic species face dual threats from unresolved taxonomic controversies and climate change. The genus *Abies*, a keystone component of alpine and subalpine ecosystems in the Northern Hemisphere, encompasses numerous species with controversial taxonomy and inadequately understood climatic response patterns. In this study, we integrated morphological and phylogenetic evidence and ecological niche modeling approaches to fill existing knowledge gaps regarding *Abies ernestii*, an endemic species found in southwest China. Key results are summarized below: (1) Morphological comparisons strongly support *A. ernestii* as a distinct species, with significant morphological differentiation from its congeneric species; phylogenetic analyses based on plastid sequences further corroborate its close phylogenetic relationship with *A. kawakamii* and *A. beshanzuensis*, rather than *A. chensiensis*. (2) The natural distribution range of *A. ernestii* is narrower than previously documented in the literature, and a newly discovered population in northern Yunnan extends its documented southern distribution boundary southward. (3) Current suitable habitats of this species are concentrated in the eastern Hengduan Mountains, where temperature seasonality-related variables (BIO11, BIO3, BIO4) exert dominant control over its distribution. (4) Future climate projections indicate a dynamic habitat shift characterized by initial expansion followed by contraction, accompanied by severe habitat fragmentation and inadequate protected area coverage. Collectively, these lines of evidence demonstrate that *A. ernestii* represents an endemic Fir with underestimated vulnerability, warranting immediate conservation prioritization.

## 1. Introduction

The conservation of mountain endemic species is under severe threat from the dual pressures of climate change and taxonomic ambiguity [1,2]. While global warming triggers habitat shifts and population fragmentation, the precise delineation of species boundaries remains a prerequisite for effective conservation intervention [3,4,5,6,7,8]. This challenge is particularly pronounced in genera with complex evolutionary histories [9,10], where morphological plasticity and hybridization obscure phylogenetic relationships, potentially leading to the misallocation of conservation resources [11,12,13]. Thus, clarifying the characteristics and ecological requirements of species through robust taxonomic delineation—by integrating morphology, molecular phylogenetics, and phylogeography—is a fundamental prerequisite for science-based conservation [14,15,16].

*Abies* Mill., the second-largest genus in the Pinaceae family, comprises approximately 50 species globally [17,18]. It originated in the Northern Hemisphere during the Late Cretaceous and underwent substantial diversification from the Miocene to the Pleistocene [19,20]. The genus has three global biodiversity hotspots: western North America, central Japan, and the Hengduan Mountains in western China [21,22]. *Abies* species serve as keystone components of subalpine coniferous forests, playing a pivotal role in shaping community structure, sustaining ecosystem resilience, and regulating hydrological and carbon cycles through complex regulatory mechanisms [23]. However, these species face severe threats from global warming-induced upward range shifts and declining low-elevation populations; human-driven habitat fragmentation further exacerbates these threats, with *Abies* species in the Hengduan Mountains facing particularly intense pressure [24,25,26,27]. Taxonomic uncertainties arising from morphological variation, natural hybridization, and inconsistent classification systems further hinder conservation initiatives for these species [19,20,28,29,30,31,32,33,34].

Within this context, *Abies ernestii*, an endemic species of the Hengduan Mountains, represents a critical understudied species with significant research gaps. It was first formally described by Alfred Rehder based on a specimen collected from Kangding, Sichuan [35]. *A. ernestii* thrives in harsh habitats, including nutrient-poor soils, steep slopes, and cold-dry climates, yet exhibits poor natural regeneration, small population sizes, and fragmented distributions [35,36]. Unlike its congeneric threatened species (e.g., *A. ziyuanensis* [37], *A. beshanzuensis* [38,39], *A. yuanbaoshanensis* [40], and *A. chensiensis* [41,42,43]), which have attracted considerable attention, *A. ernestii* lacks a robust taxonomy and comprehensive bioclimatic understanding, despite its ecological significance. Specifically, four major knowledge gaps remain unresolved: (1) Diagnostic morphology and phylogenetic position: the phylogenetic position of *A. ernestii* remains unresolved due to conflicting morphological and molecular evidence; (2) Precise distribution boundaries: its exact geographic range is poorly defined, lacking field-based and quantitative validation; (3) Core bioclimatic drivers: the key bioclimatic variables governing its distribution and its vulnerability to future climate change remain unknown; and (4) Conservation vulnerability assessment: data on population vulnerability, range contraction risks, and habitat connectivity under projected warming scenarios are lacking. Consequently, targeted conservation strategies for this species currently lack scientific support.

To address these issues, this study aims to clarify the taxonomic status of *A. ernestii* and evaluate its adaptive potential under climate change. We integrate multidisciplinary evidence to achieve four specific objectives: (I) clarify the diagnostic morphological characters and phylogenetic placement of *A. ernestii* within the genus; (II) map out the precise geographic distribution and model potential population dynamics; (III) identify core bioclimatic drivers of distribution and project habitat shifts under future climate scenarios; and (IV) assess *A. ernestii* vulnerability, including risks of range contraction and fragmentation.

## 2. Results

### 2.1. Morphology of Abies ernestii

**Figure 1:**
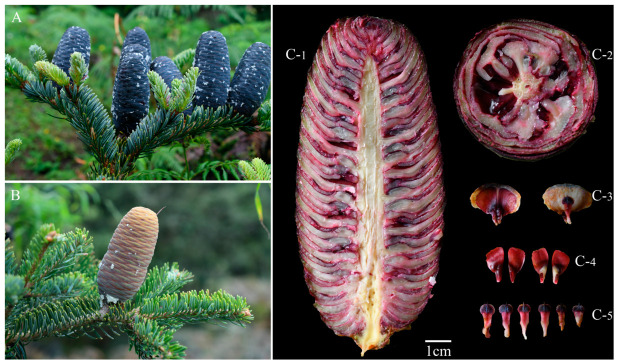
Cone morphology of *Abies ernestii*. (**A**,**B**) Cones at different maturity stages ((**A**) was taken in June; (**B**) was taken in September). (**C-1**–**C-5**) Dissection of immature cones ((**C-1**): longitudinal section; (**C-2**): cross section; (**C-3**): seed scales; (**C-4**): seeds; (**C-5**): bract scales).

*Abies ernestii* can be distinguished from its congeneric species by the following morphological traits: (1) its branchlets are initially sparsely pubescent, usually yellow, yellowish-brown, or yellowish-gray, and shedding at the mature; (2) needles on fertile branches are relatively short and possess two marginal resin ducts; (3) cones are elongate-ovoid to cylindrical, with bracts largely hidden except for apical tips that are occasionally exposed at the base. A notable taxonomic feature is the color change of cones during its development: deep blue-purple in immature stages, transitioning to pale yellow-green or brownish-green at intermediate stages, and finally darkening to light yellowish-brown or dark brown at maturity, a feature not reported in the previous literature (Figure 1).

### 2.2. Phylogeny of Abies ernestii

#### 2.2.1. Chloroplast Genome Characteristics

**Figure A1:**
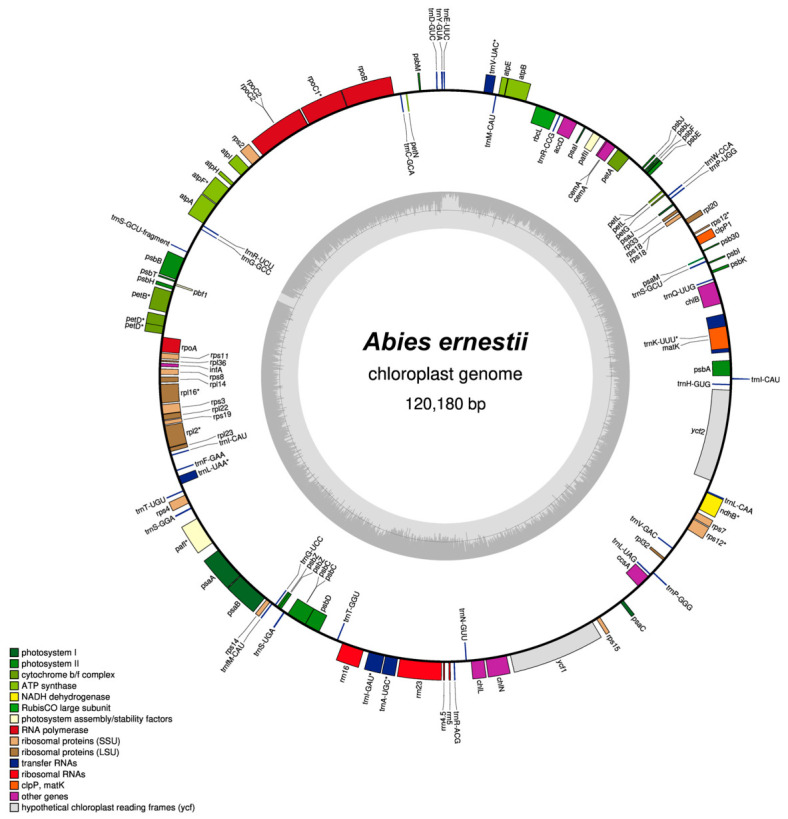
The chloroplast genome map of *Abies ernestii*. Genes shown outside the circle are transcribed clockwise, and genes inside are transcribed counterclockwise. Genes belonging to different functional groups are color-coded. The darker gray in the inner corresponds to the GC content, and the lighter gray to the AT content.

The chloroplast genome (cpDNA) of *Abies ernestii* is a 121,817 bp double-stranded, circular molecule exhibiting a typical quadripartite structure. It consists of two inverted repeat regions (IRa and IRb; 1186 bp each), a small single-copy region (SSC; 43,434 bp), and a large single-copy region (LSC; 76,314 bp). The cpDNA contains 112 genes, including 68 protein-coding genes, 34 tRNA genes, four rRNA genes, and six open reading frames (ORFs) (Table A2, Figure A1).

#### 2.2.2. Phylogenetic Inference of *Abies ernestii*

**Figure 2:**
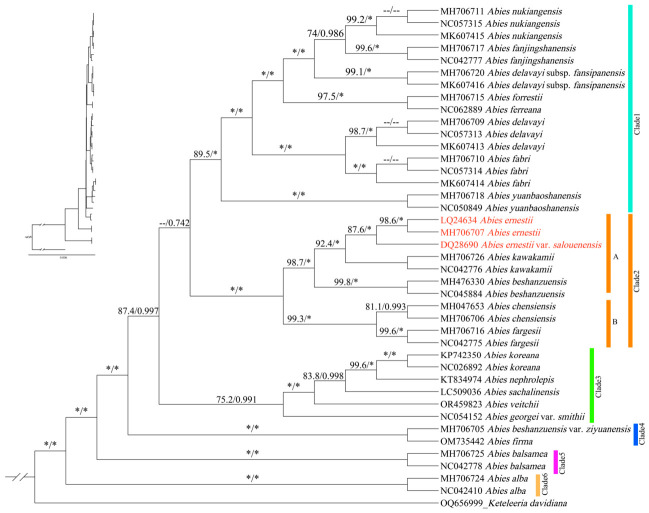
Phylogenetic relationships of the genus *Abies* based on the whole chloroplast genome sequence. The relevant numbers on the branches indicate the maximum likelihood tree support rate (ML BS) and the Bayesian tree support rate (BI PP). The dotted line (--) indicates ML BS < 50% or BI PP < 0.50. The asterisk (*) indicates ML BS = 100% or BI PP = 1.00.

Phylogenetic trees reconstructed using maximum likelihood (ML) and Bayesian inference (BI) from complete chloroplast genome sequences were topologically congruent. The 23 sampled *Abies* species formed a strongly supported monophyletic clade, which resolved into six major lineages (Figure 2). *Abies alba* and *A. balsamea* diverged earliest and occupied basal positions as sister to the remaining species. The other four clades primarily comprised species from East Asia. *A. ernestii* was nested within Clade 5 (ML BS = 100; BI PP = 1.00), a well-supported lineage that also includes *A. ernestii* var. *salouenensis*, *A. kawakamii*, *A. beshanzuensis*, *A. chensiensis*, and *A. fargesii*. Within Clade 5, *A. ernestii* and *A. ernestii* var. *salouenensis* formed a sister relationship (ML BS = 87.6; BI PP = 1.00), which in turn was sister to *A. kawakamii* (ML BS = 87.6; BI PP = 1.0). In contrast, *A. chensiensis* is resolved in a subclade (subclade B), where it clusters with *A. fargesii*.

### 2.3. Habitat Characteristics and Community Composition of Abies ernestii

*Abies ernestii* is adapted to cool temperate climates, which are characterized by a mean annual temperature ranging from 7.7 to 7.8 °C, annual precipitation between 500 and 600 mm, relative humidity > 50%, and a humid growing season. It primarily occurs on mountainous brown soils in high-elevation river valleys and slopes along major drainage systems, including the Yalong, Min, and Dadu River basins. The species exhibits a discontinuous and fragmented distribution across western Sichuan and eastern Tibet. Its core populations are concentrated below 3700 m in the middle reaches of the Yalong River, while marginal populations range from 2200 to 3300 m in the upper Min and Dadu River basins. In eastern Tibet, it is documented in Mangkang County at elevations between 3000 and 3400 m, typically along streams and in sheltered valleys. *A. ernestii* communities display a narrow elevational range (approximately 400 m), suggesting high habitat specificity. These forests feature structurally simple compositions and frequently form monospecific stands. However, they more commonly occur as mixed coniferous forests in association with other *Abies* and *Picea* species. In transition zones, *A. ernestii* co-occurs with *Pinus* and *Quercus* species, forming mixed stands that reflect disturbance or environmental gradients. Secondary succession is evident in degraded sites, where *Betula* and *Populus* species colonize, signifying disturbance-driven dynamics in community composition.

### 2.4. Potential Spatial Distribution of Abies ernestii

#### 2.4.1. Key Environmental Factors and Model Accuracy

**Figure 3:**
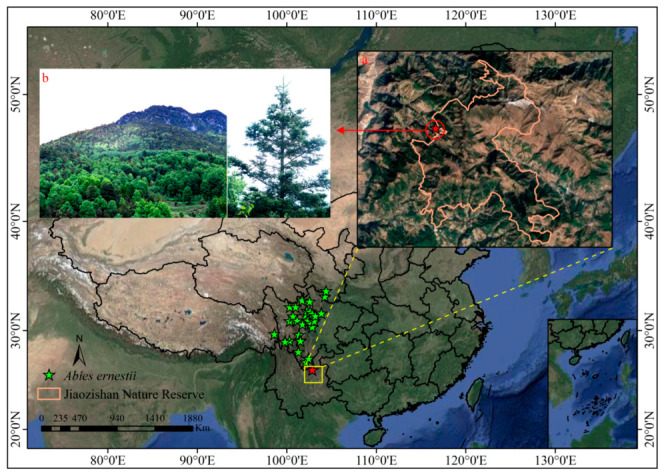
Distribution data of *Abies ernestii* in China. (**a**) The newly discovered site of *A. ernestii* in Yunnan, with the orange outline marking Jiaozi Snow Mountain Nature Reserve. (**b**) The habitat and habit of *A. ernestii* in Yunnan.

**Figure A2:**
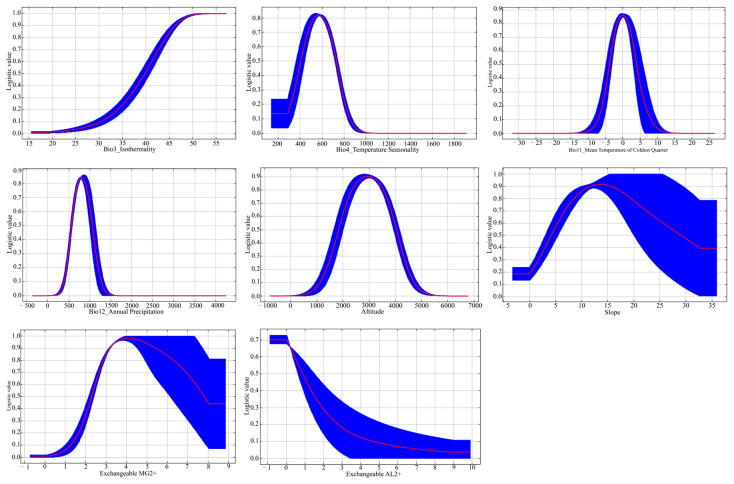
Response curves of main environmental variables.

**Figure A3:**
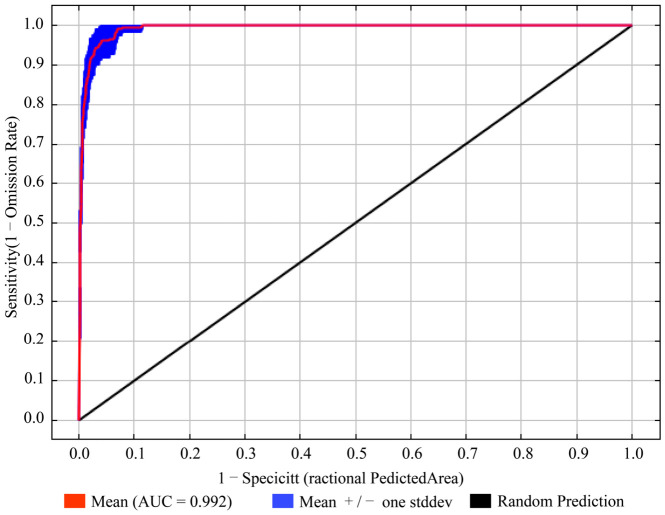
Potential habitat ROC curve of *Abies ernestii* predicted by the MaxEnt model.

After spatial filtering and collinearity analysis, 32 validated occurrence records (Figure 3) and seven key environmental predictors were retained for modeling. These included four bioclimatic variables [isothermality (bio3), temperature seasonality (bio4), mean temperature of the coldest quarter (bio11), and annual precipitation (bio12)], two topographic factors (altitude and slope), and two soil properties [exchangeable Al^3+^ (Al) and exchangeable Mg^2+^ (Mg)]. The model simulation results indicate that temperature change has the greatest influence on the distribution range of *Abies ernestii*, with the Mean Temperature of the Coldest Quarter (38.5%), Isothermality (27.9%), and Temperature Seasonality (12.1%) collectively accounting for 78.5% of the impact. Soil factors contribute an additional 16.8%, while precipitation accounts for only 1.4% (Table A3; Figure A2). MaxEnt model parameters were optimized using the ENMeval package in R. Based on evaluation metrics (Table A3; Figure A2), the optimal configuration was identified as feature classes (FC) = LQ (linear and quadratic) and regularization multiplier (RM) = 0.5. The final model was generated by averaging 10 replicate runs. The mean training AUC across replicates was 0.992 ± 0.003, suggesting strong predictive performance and high model stability (Figure A3).

#### 2.4.2. Current Distribution of Suitable Areas for *Abies ernestii*

**Figure 4:**
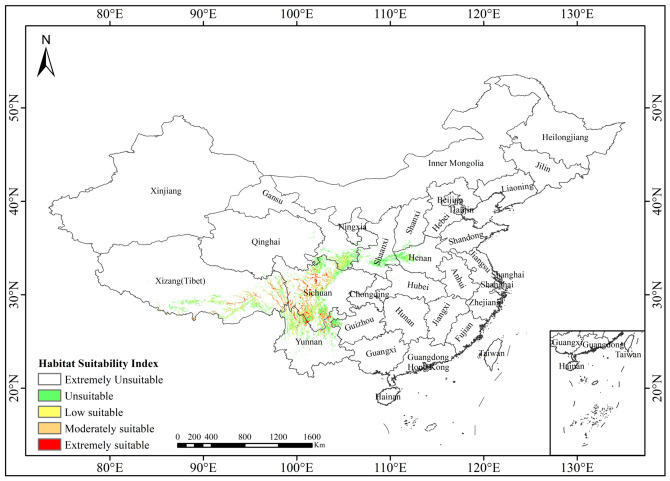
Prediction of the distribution range of *Abies ernestii* in China under current climate conditions.

Under current climatic conditions, the total area of potentially suitable habitat for *Abies ernestii* is 165,456.46 km^2^. This habitat is predominantly distributed in the eastern Hengduan Mountains region, which spans the transitional zone between the Sichuan Basin and the Qinghai–Tibet Plateau (Figure 4). Key geographic areas encompass the Ganzi Tibetan Autonomous Prefecture, the Aba Tibetan and Qiang Autonomous Prefecture, and the Liangshan Yi Autonomous Prefecture in Sichuan Province, as well as Nyingchi and Qamdo Prefectures in the Tibet Autonomous Region of China. Spatial analysis (Table 1) categorized this habitat into three suitability classes: high suitability (36,527.30 km^2^, 22.08%) under optimal climatic conditions, moderate suitability (42,580.93 km^2^, 25.74%) in suboptimal but viable environments, and low suitability (86,348.23 km^2^, 52.19%) in marginal conditions. Together, these classes account for the species’ potential range spanning heterogeneous environments.

#### 2.4.3. Changes in the Distribution Area of *Abies ernestii* Under Historical and Future Climate Scenarios

**Figure 5:**
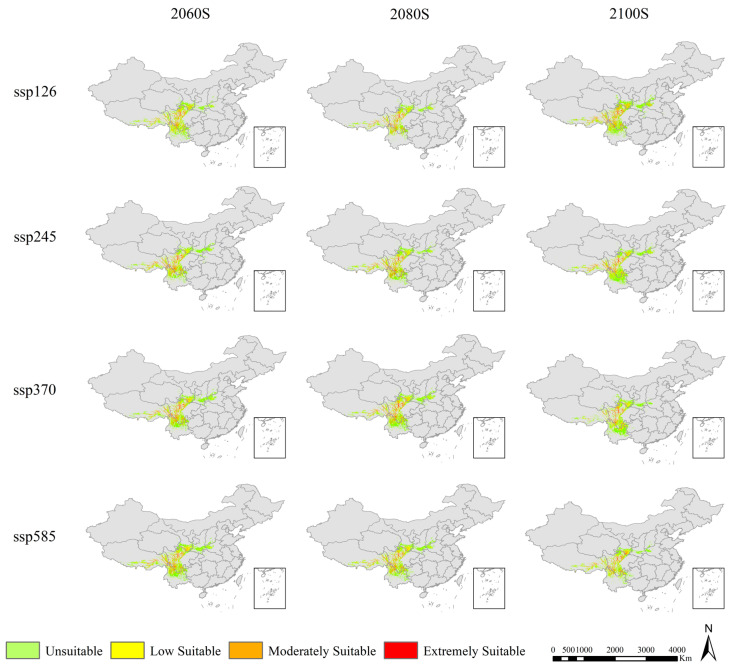
Predicted distribution of *Abies ernestii* in China under future climate conditions.

**Figure 6:**
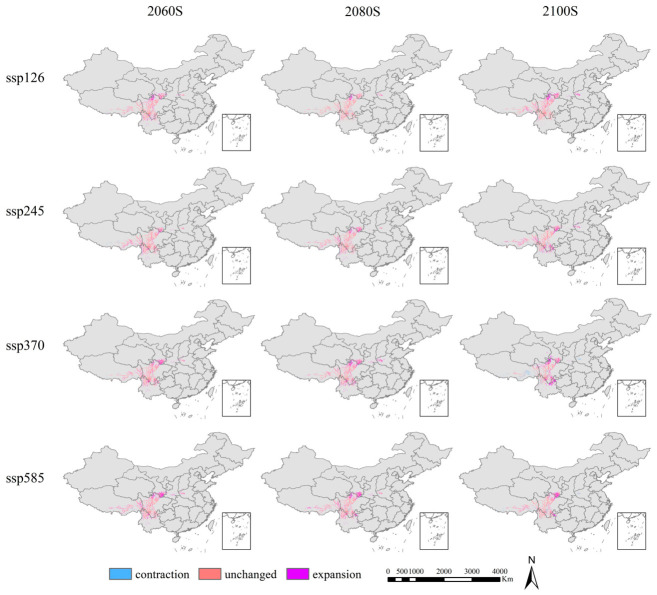
Changes in the potentially suitable areas of *Abies ernestii* from current climate conditions to future climate conditions under four SSPs.

MaxEnt model simulations under future climate scenarios (2061–2100; SSP1-2.6 to SSP5-8.5) project a unimodal trajectory in suitable habitat area for *Abies ernestii* (Figure 5, Table 1). Habitat suitability initially expands but contracts thereafter, reaching 2100 with notable inter-scenario variations in both magnitude and timing of change. Notably, SSP2-4.5 (intermediate emissions) projects the most favorable outcomes, characterized by sustained expansion of high-suitability habitat (Figure 6, Table 2). Temporal analysis reveals the 2060s as a pivotal inflection point, post which habitat expansion rates decelerate and plateau. By 2080, habitat trajectories diverge across emission pathways, stabilizing by 2100 under all scenarios as ecosystems approach a new equilibrium. However, substantial long-term contraction risks persist beyond this timeframe.

#### 2.4.4. Conservation Status of *Abies ernestii*

**Figure 7:**
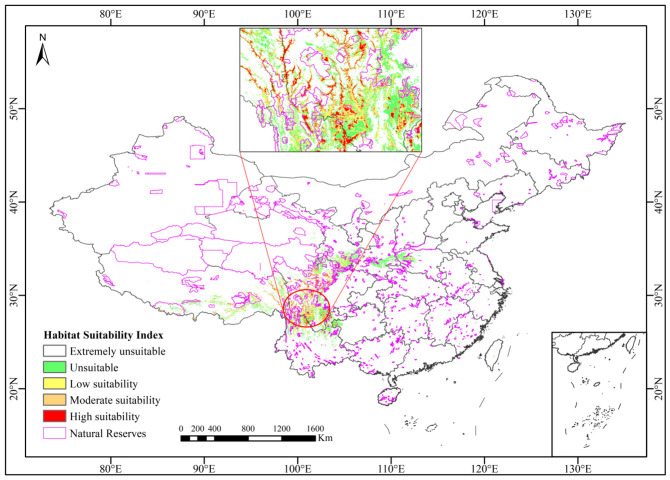
Schematic diagram of the suitability overlay of nature reserves and *Abies ernestii* habitats.

Figure 7 illustrates the spatial overlap between protected areas (comprising national and provincial nature reserves) and the habitat suitability zones of *Abies ernestii*. Spatial analysis quantifies the overlapping area as 21,688 km^2^, accounting for 13.1% of the species’ total suitable habitat (165,456 km^2^). Within these reserves, the distribution across three distinct suitability tiers: high suitability (2944 km^2^; 8.1% of total high-suitability habitat), moderate suitability (5339 km^2^; 12.5% of total moderate-suitability habitat), and low suitability (13,383 km^2^; 15.5% of total low-suitability habitat). These findings reveal that only approximately 13% of the species’ suitable range is currently under formal protection, with disproportionately low coverage (8.1%) of its most critical habitats. This disparity highlights a critical conservation gap that potentially threatens long-term population viability.

## 3. Discussion

### 3.1. Taxonomic Clarification and Phylogenetic Insights

The phylogenetic relationships and taxonomic status of *Abies ernestii* remain contentious. Early classifications by Dallimore and Jackson regarded *A. ernestii* as a synonym of *A. chensiensis*; this treatment was later modified by Liu, who treated it as a variety of *A. chensiensis* [44,45]. Subsequently, Kuan reclassified *A. ernestii* as a variety of *A. recurvata*, a taxonomic treatment followed by Eckenwalder and Farjon [18,46,47]. In contrast, Silba recognized it as a subspecies of *A. recurvata* [48]. In China, however, *A. ernestii* is widely recognized as a distinct species, encompassing two varieties, namely *A. ernestii* var. *ernestii* and *A. ernestii* var. *salouenensis* [23,35,49,50]. Shao and Xiang integrated mitochondrial and chloroplast DNA data with morphological characters and proposed reclassifying *A. ernestii* as a variety of *A. chensiensis* [30]. However, in their recent study based on whole chloroplast genome data, Shao et al. argued that *A. ernestii* is a distinct species and is closely related to *A. chensiensis* [49]. In both our BI and ML phylogenetic trees, *A. ernestii* var. *ernestii* and *A. ernestii* var. *salouenensis* are clustered together with high support, and they are more closely related to *A. kawakamii* and *A. beshanzuensis* than to *A. chensiensis*. Compared with the study by Shao et al. [49], our analyses incorporated more sequences and taxa, resulting in partially incongruent phylogenetic results. This is a relatively common phenomenon in phylogenetic analyses [51,52].

Morphologically, *A. ernestii* differs from *A. chensiensis* in marginal (vs. median) resin ducts and deep blue (vs. green-gray) immature cones. Geographically, *A. ernestii* occurs in southwestern China (vs. *A. chensiensis* in the Qinling Mountains). Given its distinct morphology, specialized ecological niche, and well-resolved phylogenetic position, *A. ernestii* warrants recognition as a distinct, independent species. The taxonomic status of *A. ernestii* var. *salouenensis* has long been taxonomically contentious; it was historically treated as a variety of *A. ernestii.* From the phylogenetic perspective, the two taxa are closely related (Figure 2), while they differ in morphology. Our study also shows that their geographic distributions are allopatric. Therefore, their relationship and taxonomic status warrant further investigation based on broader sampling.

### 3.2. Geographical Distribution and Novel Records of Abies ernestii

The actual distribution of *Abies ernestii* remains poorly documented, as historical records may overestimate its geographic range and lack rigorous empirical verification. Early studies reported that *A. ernestii* was primarily distributed in western and northern Sichuan and southeastern Tibet [46,53,54]. However, Fu et al. expanded this range in the *Flora of China* to incorporate southern Gansu, western Hubei, and northwestern Yunnan [35]. Notably, the record for northwestern Yunnan was explicitly flagged by the authors as questionable, with a question mark indicating its uncertainty. Despite this uncertainty, the *FOC*’s authoritative status led to the widespread adoption of this extended distribution in the subsequent literature [23,55]. In this study, our niche modeling results indicate that both western Hubei and northwestern Yunnan are climatically unsuitable for *A. ernestii* under current and future climate scenarios. These predictions are supported by empirical evidence. Extensive field investigations and systematic re-examination of herbarium specimens (cataloged in KUN, PE, and CDBI) uncovered no credible records or voucher specimens to confirm the natural occurrence of this species in the above regions. Integrating niche modeling results and field observational evidence, we conclude that *A. ernestii* does not occur in western Hubei and northwestern Yunnan.

Notably, our field surveys have documented the first natural population of *Abies ernestii* on Jiaozishan Mountain, Yunnan Province (26.12825118° N, 102.8318468° E; vouchers: LQ24634, LQ25517, deposited at YUKU). Located in northern Yunnan (contrary to previous biogeographic hypotheses of a northwestern distribution), this southernmost occurrence extends the species’ distribution approximately 150 km southward. This finding underscores the need to integrate ecological niche modeling with field investigations to better delineate the geographic ranges of rare conifers. However, as a peripheral population situated on the periphery of the Jiaozishan Nature Reserve of Yunnan and confined to marginal habitats (Figure 3 and Figure 4), this population faces risks of local extinction. Peripheral populations generally harbor lower genetic diversity, suffer from impaired natural regeneration—as indicated by the complete absence of seedlings and saplings—and exhibit reduced adaptive potential, rendering them exceptionally vulnerable to climate change and anthropogenic disturbances [56,57,58].

### 3.3. Changes in Suitable Habitats of Abies ernestii Under the Influence of Environmental Variables

The geographical distribution of alpine plants is primarily constrained by climatic variables, with thermal conditions playing a dominant role [59]. Multivariate analyses identified temperature dynamics as the primary driver of distribution shifts in *Abies ernestii*, with key factors including mean temperature of the coldest quarter, isothermal stability, and seasonal temperature variability. The coldest-quarter mean temperature defines the low-elevation and high-latitude survival limits. Isothermal stability mediates physiological and phenological adaptation to thermal fluctuations. Narrower winter–summer temperature differentials intensify thermal stress and reduce suitable habitat. The endangerment of fir species is primarily attributed to their specialized ecological reliance on cool, moist habitats, which are being critically undermined by escalating global temperatures [31]. Global warming is likely to shrink suitable habitats for *A. ernestii*, forcing the species to migrate to higher altitudes and causing ecological niche shifts. This process may lead to habitat fragmentation and the formation of isolated populations in mountaintop refugia, threatening long-term survival. Climate niche simulations based on future climate scenarios show that *A. ernestii* displays an overall positive response to ongoing climate warming. Under all climate scenarios, the total suitable habitat area tends to expand, with the suitable climatic space extending and core high-suitability areas remaining stable or increasing, reflecting strong climatic tolerance. These distributional shifts may be largely attributed to climate warming, rendering previously unsuitable high-elevation areas suitable. Furthermore, shifts in suitable habitat distribution exhibit pronounced dependence on scenario: habitats expand remarkably in the near future under high-emission scenarios, while moderate-emission scenarios (SSP2-4.5) provide the most optimal climatic conditions for this fir species. In contrast, the sustainability pathway scenario (SSP1-2.6) exhibits more pronounced temporal fluctuations in the extent of habitat area. Notably, different firs exhibit distinct adaptive strategies to hydrothermal gradients, reflecting their specialized niche evolution [37,42]. This interspecific variation underscores the necessity for tailored conservation strategies across their heterogeneous habitats, highlighting the imperative to preserve diverse climatic refugia to maintain fir resilience amidst global change.

### 3.4. Conservation Assessment of Abies ernestii

The conservation status of *Abies ernestii* remains ambiguous and inconsistent. Wang and Xie assessed it as Vulnerable (VU, criterion VU A2cd) in China’s Red List of Species–Volume I [60]. In contrast, the China Biodiversity Red List: Higher Plants (2020) downlisted it to Least Concern (LC) without explicit justification provided for this reassessment [61]. This discrepancy is further highlighted by the 2020 assessment of its closely related congeners—*A. chensiensis*, *A. squamata*, and *A. recurvata*—all of which remain classified as Vulnerable (VU). Notably, *A. chensiensis*, with highly similar ecological requirements, is explicitly included in China’s National List of Key Protected Wild Plants. Meanwhile, the IUCN Red List [28] still retains *A. ernestii* as Vulnerable (VU).

The predicted suitable habitat distribution of *Abies ernestii* exhibits a biogeographic pattern primarily structured by interleaved mountain and river systems. Complex mountain ranges, deep valleys, and river networks have fragmented its habitat into highly isolated ecological patches (Figure 3 and Figure 4). Studies have shown that such biogeographic barriers not only severely restrict horizontal range shifts and elevational dispersal along gradients (e.g., adaptive elevational shifts) but also strongly impede gene flow among geographically distinct populations [62]. Based on integrated evidence from field investigations, herbarium specimen re-examination, the literature collation, and niche model simulations, the present study proposes that the natural distribution range of *A. ernestii* may be narrower than previously documented. Reliable occurrences are currently confined to western and northern Sichuan, eastern Xizang, southeastern Gansu, and northern Yunnan. In contrast, credible distribution records supporting its occurrence in western Hubei and northwestern Yunnan remain insufficient, further underscoring the pivotal role of topographic fragmentation in delimiting the species’ biogeographic boundaries.

Notably, compared to sympatric fir species, *A. ernestii* has the lowest elevational distribution limit (typically below 2000 m), bringing it into proximity with human-dominated landscapes and rendering it particularly vulnerable to habitat loss and population fragmentation. Furthermore, niche model analyses demonstrate that *A. ernestii* is highly sensitive to climate change, with changes in suitable habitat area closely associated with climate scenarios. Its preference for cold-temperate climates drives poleward and upward range contractions, yet newly suitable habitats are often subject to intensified interspecific competition and pest and disease threats. Combined with the inherently slow growth and reproductive rates of firs, these factors collectively diminish their adaptive capacity and resilience to environmental perturbations. Additionally, habitat–protected area overlap analyses indicate that only 8.09% of its critical habitats are situated within protected areas, revealing substantial conservation gaps. Faced with dual threats of human disturbance and climate change, *A. ernestii* confronts an escalating survival crisis, highlighting the urgent need to upgrade its conservation status to prevent its decline into an endangered state.

## 4. Research Content and Methods

### 4.1. Morphological Research

Morphological investigations were conducted based on field observations, specimen examinations, and literature reviews. A wild population was monitored throughout its distinct phenological stages. Relevant herbarium specimens, including type materials, were reviewed and compared. Digital images from online databases, including JSTOR Global Plants (https://plants.jstor.org/), the Chinese Virtual Herbarium (http://www.cvh.ac.cn/), the Plant Photo Bank of China (https://ppbc.iplant.cn/), and the Global Biodiversity Information Facility (https://www.gbif.org/occurrence/download/0015226-260430073515954 accessed on 6 June 2025), were extensively reviewed. Fresh samples and dried specimens were analyzed using a stereomicroscope. Key diagnostic organs—specifically cones, leaves, and resin ducts—were selected for quantitative analysis. Measurements were taken using a metric ruler and a vernier caliper (accurate to 0.01 mm) to record parameters such as length, width, and thickness, thereby establishing morphological benchmarks.

### 4.2. Molecular Systematics

#### 4.2.1. DNA Extraction, Sequencing, and Assembly

Healthy leaf samples were collected and dried on silica gel before being sequenced by Novogene on the Illumina NovaSeq 6000 platform using 150 bp double-end sequencing. GetOrganelle v1.7.5 was used for assembly [63]; the GeSeq online tool (https://chlorobox.mpimp-golm.mpg.de/geseq.html, accessed on 6 June 2025)) was used for gene annotation [64], and Geneious Prime v2022.2.4 was used for manual correction [65].

#### 4.2.2. Phylogenetic Analysis

Maximum likelihood (ML) and Bayesian inference (BI) analyses were conducted using PhyloSuite v1.2.2 [66] based on 42 chloroplast genome sequences (including 41 *Abies* species and the outgroup *Keteleeria davidiana*). To improve the reliability of phylogenetic analyses, we supplemented the species dataset of Shao et al. [49,50] with additional *Abies* taxon (Table A1) and independently field-collected individuals of *A. ernestii* (LQ24634) and *A. ernestii* var. *Salouenensis* (DQ28690). This modification aimed to maintain research comparability while better reflecting actual evolutionary relationships in the analytical results. Forty of these sequences were downloaded from GenBank (their accession numbers are listed in Table A1). Sequence alignment was performed with MAFFT [67], and the resulting alignment was trimmed using trimAl [68]. The optimal evolutionary models were selected based on the AIC and BIC criteria: TIM3 + F + I for the BI analysis and K2P + I for the ML analysis [69]. The BI analysis was run using MrBayes 3.2.6 [70], and the ML analysis was run using IQ-TREE [71], both within PhyloSuite v1.2.2 [66].

### 4.3. Spatial Distribution Study Based on MaxEnt

#### 4.3.1. Collection of Species Distribution Data and Environmental Variable Data

Species distribution records were compiled from field surveys, the literature, and public databases. Following verification to remove invalid, duplicate, and outlier records, valid data were processed for spatial autocorrelation using ENMTools [72]. Environmental variables included topographic data, 19 bioclimatic layers from WorldClim (https://www.worldclim.org/; versions 2.1, covering contemporary climate, paleoclimate, and future climate scenarios under four general circulation models: ACCESS-CM2, BCC-CSM2-MR, CMCC-CM2, and EC-Earth3-Veg) [73], and 37 soil variables (http://globalchange.bnu.edu.cn/) [74]. To mitigate multicollinearity, Pearson correlation tests (|r| ≥ 0.8) were used to retain variables with the highest contribution rates, resulting in eight variables for the MaxEnt analysis [75,76,77].

#### 4.3.2. Model Running, Spatial Pattern Changes, and Gap Analysis

Given that the MaxEnt model performance is highly sensitive to parameter settings, specifically feature classes (FC) and the regularization multiplier (RM), careful tuning is essential to prevent overfitting [78,79]. In this study, the ENMeval package in R was employed to optimize these parameters. The tested RM values ranged from 0.5 to 4.0 in increments of 0.5, crossed with six feature class combinations (L, LQ, H, LQH, LQHP, LQHPT). The optimal parameter set was selected based on two criteria: an omission rate of less than 5% and the lowest Akaike Information Criterion corrected for small sample sizes (AICc) value [80]. MaxEnt version 3.4.4 was used to predict potentially suitable habitats using the optimized parameters. Model settings included 75% of occurrence records for training and 25% for validation, with 10 replicate runs of 5000 iterations each. Model accuracy was evaluated using the area under the receiver operating characteristic (ROC) curve (AUC), and the Jackknife method was used to assess the contribution of environmental variables. For future climate scenarios, we averaged simulated distribution layers derived from multiple General Circulation Models (GCMs) to generate consensus predictions. These predictions were then imported into ArcGIS 10.8.2 and classified into five habitat suitability levels using the natural breaks (Jenks) method [81]. The SDMToolbox extension in ArcGIS 10.8.2 was subsequently used to evaluate changes in suitable habitat areas under projected future climatic conditions [82,83]. Finally, we overlaid the predicted habitat distribution map with vector data on national nature reserves, sourced from the China National Nature Reserve Specimen Resource Platform, to calculate the proportion of suitable habitat currently under protection.

## 5. Conclusions

Phylogenetic analyses firmly confirm *Abies ernestii* as an independent species and resolve its precise phylogenetic position. Concurrent morphological observations and analyses have yielded additional new findings, elucidating specific, distinctive morphological transitions throughout cone development. We refine the distribution range of this species within the eastern Hengduan Mountains, where a newly discovered population in Yunnan extends its southern distribution boundary by approximately 150 km and rectifies historical misidentifications. Niche modeling analyses highlight the Mean Temperature of the Coldest Quarter (Bio11) and Annual Precipitation (Bio12) as the primary climatic determinants of its suitable habitat. Critically, only 8.09% of its highly suitable habitats lie within existing protected areas, revealing a significant conservation gap. To address this gap, we propose three priority conservation actions: (1) Revise the Conservation Status: Given its high vulnerability to climate change and the narrowly restricted distribution of extant populations, we recommend uplisting A. ernestii on the IUCN Red List; (2) Strengthen Habitat Protection: Expand protected area networks to incorporate climate refugia and marginal populations; (3) Deepen Genetic Research: Prioritize studies on the genetic diversity of peripheral populations to guide the formulation of adaptive conservation strategies.

## Figures and Tables

**Table 1:** Statistics of suitable areas for *Abies ernestii* in different periods.

		Low Suitable	Moderately Suitable	Extremely Suitable	Summary
Current		86,348.23	42,580.93	36,527.30	165,456.46
2060	ssp126	117,878.18	55,843.28	35,965.48	209,686.94
	ssp245	96,120.75	52,715.16	50,725.99	199,561.90
	ssp370	109,651.92	56,799.86	50,654.39	217,106.17
	ssp585	124,732.66	57,901.57	52,582.21	235,216.45
2080	ssp126	89,487.95	44,528.17	40,103.14	174,119.25
	ssp245	98,596.52	45,721.14	55,827.57	200,145.23
	ssp370	114,351.07	57,305.64	53,608.92	225,265.63
	ssp585	121,189.09	51,959.79	47,844.17	220,993.06
2100	ssp126	118,200.82	52,957.05	42,580.66	213,738.54
	ssp245	111,057.72	47,017.47	41,820.55	199,895.74
	ssp370	115,083.05	44,439.11	36,387.39	195,909.56
	ssp585	98,844.54	47,428.69	39,427.85	185,701.08

**Table 2:** Change rate of suitable areas for *Abies ernestii* over different periods.

		Expansion	Unchanged	Contraction
Current-2060	ssp126	25.69%	68.13%	6.18%
	ssp245	21.41%	73.46%	5.13%
	ssp370	26.79%	68.93%	4.28%
	ssp585	32.38%	63.29%	4.33%
Current-2080	ssp126	13.94%	76.18%	9.88%
	ssp245	22.39%	71.29%	6.32%
	ssp370	30.67%	63.23%	6.09%
	ssp585	29.63%	64.01%	6.35%
Current-2100	ssp126	25.65%	69.80%	4.55%
	ssp245	25.37%	64.55%	10.08%
	ssp370	29.67%	53.33%	17.01%
	ssp585	21.15%	67.04%	11.80%

## Data Availability

The original contributions presented in this study are included in the article. Further inquiries can be directed to the corresponding authors.

## Appendix A

This appendix contains the tables related to this research.

**Table A1:** Species sequence information downloaded from GenBank.

Family	Genus	Species	GenBank	Length
Pinaceae	*Abies*	*Abies alba* Mill.	MH706724	121,243 bp
Pinaceae	*Abies*	*Abies alba* Mill.	NC042410	121,243 bp
Pinaceae	*Abies*	*Abies ziyuanensis* L. K. Fu and S. L. Mo	MH706705	121,274 bp
Pinaceae	*Abies*	*Abies balsamea* (L.) Mill.	NC042778	121,574 bp
Pinaceae	*Abies*	*Abies balsamea* (L.) Mill.	MH706725	121,574 bp
Pinaceae	*Abies*	*Abies beshanzuensis* M. H. Wu	NC045884	121,399 bp
Pinaceae	*Abies*	*Abies beshanzuensis* M. H. Wu	MH476330	121,399 bp
Pinaceae	*Abies*	*Abies chensiensis* Tiegh.	MH047653	121,498 bp
Pinaceae	*Abies*	*Abies chensiensis* Tiegh.	MH706706	121,795 bp
Pinaceae	*Abies*	*Abies delavayi* Diels	NC057313	120,141 bp
Pinaceae	*Abies*	*Abies delavayi* Diels	MH706709	120,141 bp
Pinaceae	*Abies*	*Abies delavayi* Diels	MK607413	120,142 bp
Pinaceae	*Abies*	*Abies delavayi* subsp. *fansipanensis* (Q.P. Xiang, L.K. Fu, and Nan Li) Rushforth	MH706720	120,093 bp
Pinaceae	*Abies*	*Abies delavayi* subsp. *fansipanensis* (Q.P. Xiang, L.K. Fu, and Nan Li) Rushforth	MK607416	120,094 bp
Pinaceae	*Abies*	*Abies ernestii* Rehder	MH706707	121,841 bp
Pinaceae	*Abies*	*Abies fabri* (Mast.) Craib	NC057314	120,027 bp
Pinaceae	*Abies*	*Abies fabri* (Mast.) Craib	MH706710	120,027 bp
Pinaceae	*Abies*	*Abies fabri* (Mast.) Craib	MK607414	120,027 bp
Pinaceae	*Abies*	*Abies fanjingshanensis* W. L. Huang, Y. L. Tu, and S. Z. Fang	NC042777	120,057 bp
Pinaceae	*Abies*	*Abies fanjingshanensis* W. L. Huang, Y. L. Tu, and S. Z. Fang	MH706717	120,057 bp
Pinaceae	*Abies*	*Abies fargesii* Franch.	NC042775	121,799 bp
Pinaceae	*Abies*	*Abies fargesii* Franch.	MH706716	121,799 bp
Pinaceae	*Abies*	*Abies ferreana* Bordères and Gaussen	NC062889	120,049 bp
Pinaceae	*Abies*	*Abies firma* Siebold and Zucc.	OM735442	121,395 bp
Pinaceae	*Abies*	*Abies forrestii* Coltm.-Rog.	MH706715	120,022 bp
Pinaceae	*Abies*	*Abies georgei* var. *smithii* (Viguié and Gaussen) W. C. Cheng and L. K. Fu	NC054152	121,191 bp
Pinaceae	*Abies*	*Abies kawakamii* (Hayata) T. Itô	NC042776	121,290 bp
Pinaceae	*Abies*	*Abies kawakamii* (Hayata) T. Itô	MH706726	121,290 bp
Pinaceae	*Abies*	*Abies nephrolepis* (Trautv. ex Maxim.) Maxim.	KT834974	121,336 bp
Pinaceae	*Abies*	*Abies nukiangensis* W. C. Cheng and L. K. Fu	NC057315	120,017 bp
Pinaceae	*Abies*	*Abies nukiangensis* W. C. Cheng and L. K. Fu	MH706711	120,017 bp
Pinaceae	*Abies*	*Abies nukiangensis* W. C. Cheng and L. K. Fu	MK607415	120,017 bp
Pinaceae	*Abies*	*Abies koreana* E. H. Wilson	NC026892	121,373 bp
Pinaceae	*Abies*	*Abies koreana* E. H. Wilson	KP742350	121,373 bp
Pinaceae	*Abies*	*Abies veitchii* Lindl.	OR459823	121,403 bp
Pinaceae	*Abies*	*Abies yuanbaoshanensis* Y. J. Lu and L. K. Fu	NC050849	121,795 bp
Pinaceae	*Abies*	*Abies yuanbaoshanensis* Y. J. Lu and L. K. Fu	MH706718	121,795 bp
Pinaceae	*Abies*	*Abies sachalinensis* (F.Schmidt) Mast.	LC509036	121,337 bp
Pinaceae	*Keteleeria*	*Keteleeria davidiana* (C. E. Bertrand) Beissn.	OQ656999	117,808 bp

**Table A2:** List of genes encoded in *Abies ernestii* chloroplast genomes.

Groups of Genes	Name of Genes
Ribosomal RNAs	rrn4.5, rrn5, rrn16, rrn23
Transfer RNAs	trnA-UGC *, trnC-GCA, trnD-GUC, trnE-UUC, trnF-GAA, trnfM-CAU, trnG-GCC *, trnG-UCC, rrnH-GUG, trnI-CAUx2, trnI-GAU *, trnK-UUU *, trnL-CAA, trnL-UAA *, trnL-UAG, trnM-CAU, trnN-GUU, trnP-GGG, trnP-UGG, trnQ-UUG, trnR-ACG, trnR-CCG, trnR-UCU, trnS-GCUx2, trnS-GGA, trnS-UGA, trnT-GGU, trnT-UGU, trnV-GAC, trnV-UAC *, trnW-CCA, trnY-GUA
Subunits of photosystem I	psaA, psaB, psaC, psaI, psaJ, psaM, psaM
Subunits of photosystem II	psbA, psbB, psbC, psbD, psbE, psbF, psbH, psbI, psbJ, psbK, psbL, psbM, psbN, psbT, psbZ
Subunits of the cytochrome b/f complex	petA, petB *, petD *, petG, petL, petN
Subunits of ATP synthase	atpA, atpB, atpE, atpF *, atpH, atpI
Proteins of the large ribosomal subunit	rpl2 *, rpl14, rpl16 *, rpl20, rpl22, rpl23, rpl32, rpl33, rpl36
Proteins of the small ribosomal subunit	rps2, rps3, rps4, rps7, rps8, rps11, rps12 **, rps14, rps15, rps18, rps19
Large subunit of RuBisco	rbcL
Subunits of RNA polymerase	rpoA, rpoB, rpoC1 *, rpoC2
Conserved hypothetical chloroplast reading frames	ycf1, ycf2, ycf3 **, ycf4, ycf12x2
ATP-dependent protease subunit P	cemA
Chloroplast envelope membrane protein	clpP
Chlorophyll biosynthesis	chlB, chlL, chlN
Miscellaneous proteins	accD, ccsA, infA, matK

Notes: * represents the gene contains one intron, ** represents the gene contains two introns, x2 represents the gene has two copies.

**Table A3:** Contribution rate of environmental variables.

Variable	Percent Contribution	Permutation Importance
Bio_11	38.5	17.1
Bio_3	27.9	9.9
Bio_4	12.1	50
Mg	11.5	1.1
Alt	5.3	15.6
Slope	1.9	0
Bio_12	1.4	2.3
Al	1.4	3.9

## Appendix B

This appendix contains the figure related to this research.
